# PP06 Incentives To Incorporate Innovation Into Care Delivery Processes: A Scoping Review And SWOT Analysis

**DOI:** 10.1017/S026646232400179X

**Published:** 2025-01-07

**Authors:** Madeleine Haig, Caitlin Main, Panos Kanavos

## Abstract

**Introduction:**

This study investigated the various incentives employed in Organisation for Economic Co-operation and Development (OECD) and European Economic Area (EEA) countries to enhance access to innovative medical technologies. The literature review encompasses real and theoretical models, offering an overview of strategies to bridge the gap between innovation and access. A strengths, weaknesses, opportunities, and threats (SWOT) analysis was conducted to analyze incentives in health system and therapeutic area context.

**Methods:**

The review methodically examined peer-reviewed articles, reports, and policy documents published between 2000 and 2023. Databases searched include PubMed, Scopus, Web of Science, and EconLit. Grey literature was searched from international organizations’ websites, including the World Bank, World Health Organization (WHO), OECD, Pan American Health Organization (PAHO), and European Commission. Inclusion criteria focused on relevance of financial and non-financial mechanisms to effective implementation of innovative medical technologies, and their application within OECD and EEA countries. COVID-19 research, vaccines, cost-effectiveness studies, and studies that did not discuss implementation were excluded. A SWOT analysis was utilized to categorize the mechanisms by therapeutic area and health system design.

**Results:**

The review identified diverse mechanisms, including reinsurance, impact bonds, outcomes-based agreements, annuity payments, and risk-sharing agreements. Financial mechanisms, such as outcomes-based agreements, were prominent but highlighted implementation obstacles, including a lack of data infrastructure capable of linking outcomes to payments, which ultimately undermines the effectiveness of these strategies. Non-financial mechanisms, such as population health management, were also identified. The effectiveness varied, with some models showing significant improvement in technology accessibility, while others faced implementation and affordability challenges. Comparative analysis highlighted differences in efficacy dependent on the therapeutic area and type of health system in which the incentive is applied.

**Conclusions:**

The review underscores multifaceted approaches to improve access to innovative medical technologies. While financial incentives play a crucial role, non-financial strategies are also vital. This study provides insights into which incentives are most effective in certain health systems and therapeutic areas. Policymakers can benefit from these insights, leveraging successful models and addressing challenges to ensure equitable access to medical innovations.

